# Targeting Proteasomes in Cancer and Infectious Disease: A Parallel Strategy to Treat Malignancies and Microbes

**DOI:** 10.3389/fcimb.2022.925804

**Published:** 2022-07-07

**Authors:** James J. Ignatz-Hoover, Elena V. Murphy, James J. Driscoll

**Affiliations:** ^1^ Division of Hematology & Oncology, Department of Medicine, Case Western Reserve University, Cleveland, OH, United States; ^2^ Adult Hematologic Malignancies & Stem Cell Transplant Section, Seidman Cancer Center, University Hospitals Cleveland Medical Center, Cleveland, OH, United States; ^3^ Case Comprehensive Cancer Center, School of Medicine, Case Western Reserve University, Cleveland, OH, United States; ^4^ Case Western Reserve University, Department of Biochemistry, Cleveland, OH, United States

**Keywords:** proteasome, cancer, infection, myeloma, malaria, tuberculosis

## Abstract

Essential core pathways of cellular biology are preserved throughout evolution, highlighting the importance of these pathways for both bacteria and human cancer cells alike. Cell viability requires a proper balance between protein synthesis and degradation in order to maintain integrity of the proteome. Proteasomes are highly intricate, tightly regulated multisubunit complexes that are critical to achieve protein homeostasis (proteostasis) through the selective degradation of misfolded, redundant and damaged proteins. Proteasomes function as the catalytic core of the ubiquitin-proteasome pathway (UPP) which regulates a myriad of essential processes including growth, survival, differentiation, drug resistance and apoptosis. Proteasomes recognize and degrade proteins that have been marked by covalently attached poly-ubiquitin chains. Deregulation of the UPP has emerged as an essential etiology of many prominent diseases, including cancer. Proteasome inhibitors selectively target cancer cells, including those resistant to chemotherapy, while sparing healthy cells. Proteasome inhibition has emerged as a transformative anti-myeloma strategy that has extended survival for certain patient populations from 3 to 8 years. The structural architecture and functional activity of proteasomes is conserved from *Archaea* to humans to support the concept that proteasomes are actionable targets that can be inhibited in pathogenic organisms to improve the treatment of infectious diseases. Proteasomes have an essential role during all stages of the parasite life cycle and features that distinguish proteasomes in pathogens from human forms have been revealed. Advancement of inhibitors that target *Plasmodium* and *Mycobacterial* proteasomes is a means to improve treatment of malaria and tuberculosis. In addition, PIs may also synergize with current frontline agents support as resistance to conventional drugs continues to increase. The proteasome represents a highly promising, actionable target to combat infectious diseases that devastate lives and livelihoods around the globe.

## Overview of Proteasome Complexes

**Figure 1:**
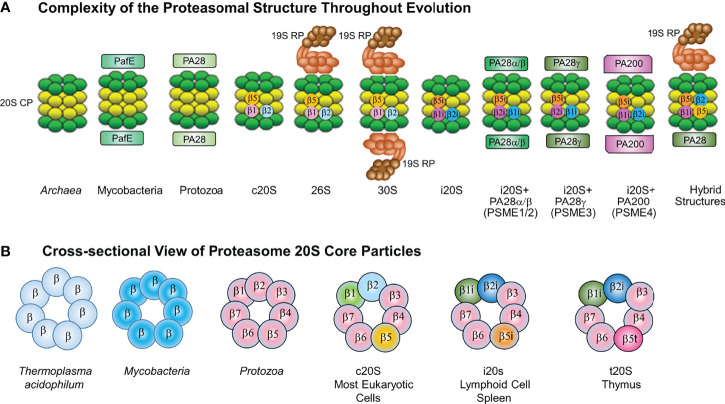
**(A)** Complexity of the Proteasome Structure Throughout Evolution. The constitutive 20S proteasome is assembled in a (green) and b (yellow) heptameric rings. Constitutive 20S proteasomes (c20S) contain the catalytic subunits b1, b2 and b5. The 20S immunoproteasome (i20S) catalytic core contains three inducible b subunits named b1i, b2i and b5i. immunoproteasomes As previously described, intracellular proteasomes can exist in multiple different forms (Gomes, 2013). Both c20S and i20S proteasomes are found with or without regulators. The c20S is capped at one or both ends by a 19S RP (light brown) to form 26S or 30S proteasomes. The i20S proteasome is capped by either the PA28ab or PA28g complex at either one or both ends. Hybrid proteasome complexes are also found when the catalytic core is simultaneously associated with 19S RP and another type of regulator, e.g., PA28ab, PA28g, PA200. In the cytoplasm ECM29 and PSMF1 can modify the assembly or activity of the proteasome; while in the nucleus PA200 can regulate proteasome activity. **(B)** Cross-sectional View of Proteasome 20S Core Particles. Shown is a cross-sectional side view of 20S proteasomes showing the evolutionarily conserved barrel-like (a7b7b7a7) structure in eubacterial and eukaryotic proteasomes. Archaea and Mtb proteasomes are depicted as having seven identical b subunits, whereas eukaryotic proteasomes have seven different b subunits. In lymphoid cells and spleen, constitutive 20S proteasome catalytic subunits b1, b2 and b5 are substituted by three inducible b subunits named b1i, b2i and b5i to generate 20S proteasomes. 26S proteasomes exist with either one or two 19S caps, immunoproteasomes containing one or two 11S caps, proteasomes containing the 20S proteasome with one or two PA200 caps (in the nucleus only), and hybrid proteasomes which contain different combinations of 20S and activators.

Protein homeostasis (proteostasis) is pivotal for cell viability and is governed by precisely regulated processes that balance protein synthesis and degradation (Cromm and Crews, 2017). Cells utilize protein clearance mechanisms, e.g., the ubiquitin (Ub)-proteasome pathway (UPP), autophagy, and aggresomes, to regulate essential biological processes, eliminate misfolded, unfolded, denatured and redundant proteins and modulate the levels of regulatory proteins that govern cell cycle progression and the circadian rhythm (Kopito, 2000; Ciechanover, 2005; Finley, 2009; Dikic, 2017). The UPP is responsible for the majority of protein degradation within eukaryotic cells (Finley, 2009). Protein substrates are marked for turnover through covalent linkage of poly-Ub chains (Ciechanover, 2005). Ubiquitinated substrates are recognized by a 26S (~2.5MDa) ATP+Ub-dependent complex that hydrolyzes substrates into peptides and recycles free Ub. Eukaryotic cells contain high molecular weight (HMW), cylindrical ribonucleoprotein particles (RNPs) composed of a family of proteins with molecular masses of 19-36 kilodaltons. Numerous groups had described a non-lysosomal, multicatalytic proteinase (MCP) that exhibited peptide-hydrolyzing activity at neutral-alkaline pH against N-blocked tripeptide substrates with an Arg, Phe or Glu residue adjacent to 4-MCA or 2-naphthylamide leaving groups (Wilk and Orlowski, 1980; Wilk and Orlowski, 1983; Dahlmann et al., 1983; Ray and Harris, 1985). Subsequently, Falkenberg et al. demonstrated by using electron microscopy, western blotting, RNA analysis and proteinase assays, that the RNPs and MCPs were structurally and immunologically-related entities (Falkenburg et al., 1988). Later studies showed that the 20S MCP - *the proteasome* - is in fact incorporated into the 26S ATP+Ub-dependent proteasome complex (Eytan et al., 1989; Driscoll and Goldberg, 1990). The 26S proteasome complex is assembled in an ATP-dependent manner by capping the 20S catalytic core particle (CP) at either or both ends with 19S regulatory particles (RP) that confer ATP and Ub-dependence (**Figure 1**).

Proteasomes are conserved from *Archaea* to humans, are found within the cytoplasm and nucleus and all forms observed thus far contain a 20S CP (Groll et al., 1997; Voges et al., 1999; Maupin-Furlow, 2012). In contrast to the 26S complex, the 20S proteasome can recognize and degrade unfolded or unstructured protein substrates in a Ub-independent manner (Driscoll and Goldberg, 1989; Matthews et al., 1989). The constitutive 20S proteasome (c20S) CP is arranged as 4 stacked heptameric rings in an α_α_β_α_β_α_α_7_ barrel-shaped manner (Groll et al., 1997; Voges et al., 1999; Ciechanover, 2005; Finley, 2009; Maupin-Furlow, 2012). The 2 distal rings are identical and contain 7 different α subunits, whereas the 2 central rings contain 7 different β subunits, of which β1, β2, and β5 exert catalytic activities. Proteasomes are a family of N-terminal hydrolases and each catalytic subunit has been ascribed a typical activity profile. The β1 subunit cleaves predominantly after acidic amino acids (caspase-like activity), β2 cleaves after basic residues (trypsin-like activity), and the β5 subunit after hydrophobic amino acids (chymotrypsin-like/ChT-like) (Dahlmann et al., 1989; Dick et al., 1998; Gallastegui and Groll, 2010). Proteasomal hydrolysis of intracellular proteins also generates peptides that function as antigens bound to the major histocompatibility complex (MHC) class I molecule. In lymphoid cells and cells exposed to IFN-γ, the β1, β2, and β5 subunits within c20S proteasomes are replaced by their inducible counterparts β1i, β2i, and β5i, leading to immunoproteasome (i20S) formation (Brown et al., 1991; Driscoll and Finley, 1992; Kloetzel, 2001). i20S proteasomes exhibit catalytic activities distinct from c20S proteasomes, with lower caspase-like and greater ChT-like activity (Driscoll et al., 1993). Presentation of MHC class I antigens is favored in cells that harbor i20S proteasomes to shape the repertoire of immunodominant epitopes.

## Targeting the Proteasome in Cancer Treatment

Proteasomal inhibition leads to the accumulation of Ub-protein conjugated proteins that triggers endoplasmic reticulum (ER) stress, activates the unfolded protein response (UPR) and culminates in cell death. PIs are validated, safe and effective anti-cancer drugs (Orlowski and Kuhn, 2008; Dick and Fleming, 2010; Kisselev et al., 2012; Fricker, 2020). Bortezomib (Millennium-Takeda) is a boronate dipeptide aldehyde and first FDA-approved PI for MM. Bortezomib acts predominantly on the proteasomal ChT-L activity and displays potent inhibitory activity against the *β*1i (IC_50_ = 5.5 nM), *β*5c (IC_50_ = 7 nM) and *β*5i (IC_50_ = 3.3 nM) subunits (**Table 1**). However, dose-dependent peripheral neuropathy may limit clinical utility. In addition, bortezomib has limited activity against solid tumors since adequate dosing cannot be reached to overcome poor tissue penetration and rapid clearance from peripheral blood (Orlowski and Kuhn, 2008; Dick and Fleming, 2010; Kisselev et al., 2012; Fricker, 2020).

**Table 1:** Fda-approved and investigational proteasomal inhibitors for the treatment of human disease.

DISEASE	STRUCTURE			STATUS	ACTIVE SITE	DISEASE TARGET/ DISEASE MODEL
**ONCOLOGY**						
Bortezomib (Orlowski and Kuhn, 2008; Dick and Fleming, 2010; Kisselev et al., 2012; Fricker, 2020)	Dipeptidyl boronate			FDA-Approval	β5>β1>>β2	MM, MCL, DLBCL
				RRMM (2003)		
				MM (2007)		
				Previously untreated MM (2008)		
				SC injection for all approved indications (2012)		
				MCL- with at least one prior tx (2007)		
Carfilzomib (Kuhn et al., 2007; Huber and Groll, 2012; Herndon et al., 2013; Park et al., 2018; Jayaweera et al., 2021)	Tetrapeptide epoxyketone			FDA-Approval	β5>β1>β2	MM
				Advanced MM (2012)		
				Relapsed MM (2015)		
				RRMM (2016)		
Ixazomib (Kumar et al., 2014; Al-Salama et al., 2017)	Dipeptidyl boronate, oral			FDA-Approval	β5>β1>>β2	Oral agent, MM
				RRMM (2015)		
Marizomib (Huber and Groll, 2012; Park et al., 2018; Fricker, 2020)	β-lactone			FDA orphan status	β5>β2>β1	MM, crosses BBB
(Salinosporamide A, NPI-0052)				MM (2013)		
Delanzomib (Huber and Groll, 2012; Park et al., 2018; Fricker, 2020)	C-terminal boronic acid peptide (phenylpyridine)				β5>β1>>β2	MM
(CEP-18770)						
ONX-0912 (Johnson et al., 2017; Schmidt et al., 2018; Xi et al., 2019)				Orphan status	β5>β1>>β2	MM, WM
(Oprozomib, PR-047)	oral			NCT02072863		
Lactacystin (Orlowski and Kuhn, 2008; Dick and Fleming, 2010; Kisselev et al., 2012; Fricker, 2020)	β-lactone			Pre-clinical	β5>β1>>β2	Cancers, MM
TMC-95A, B, C, D (Koguchi et al., 2000)	*Apiospora* broth			Pre-clinical	β5>β1>>β2	TMC-95A and diastereomers (B-D) exhibited cytoxicity against human HCT-116 colon
**AUTOIMMUNE DISEASES**						
ONX-0914 (Kuhn et al., 2007; Huber and Groll, 2012; Herndon et al., 2013; Johnson et al., 2017; Park et al., 2018; Schmidt et al., 2018; Xi et al., 2019; Jayaweera et al., 2021)	Tripeptide Epoxyketone			Investigational	β5i>β1i>β2i	Experimental/murine autoimmune, rheumatoid arthritis, encephalomyelitis, inflammatory bowel disease, and GVHD models
(PR-957)						
KZR-504 (Johnson et al., 2017; Xi et al., 2019)	Dipeptide Epoxyketone			Investigational	β1i>β5i>β2	RA, SLE
KZR-616 (Johnson et al., 2017; Xi et al., 2019)	Tetrapeptide Epoxyketone			NCT03393013	β5i>β1i>β2i	SLE with and without Lupus Nephritis
				NCT04033926		Polymyositis or Dermatomyositis
				NCT04039477		Active Autoimmune Hemolytic Anemia or ITP
**TUBERCULOSIS**						
GL5 (Totaro et al., 2017)	Oxathiazole-2-one derivative			Pre-clinical	β5	Mtb20S-selective Kills non-replicating Mtb under NO stress Selective suicide substrate inhibitor of Mtb 20S OG proteasomes Cyclocarbonylates active site Thr >1,000-fold more effective against Mtb than human proteasomes
HT1171 (Totaro et al., 2017)	Oxathiazole-2-one derivative			Pre-clinical	β5	Mtb20S-selective Kills non-replicating Mtb under NO stress Selective suicide substrate inhibitor of Mtb 20S OG proteasomes Cyclocarbonylates the active site Thr >1,000-fold more effective against Mtb than human proteasomes
HT1171 (Totaro et al., 2017)	Oxathiazole-2-one derivative			Pre-clinical	β5	Mtb20S-selective Kills non-replicating Mtb under NO stress Selective suicide substrate inhibitor of Mtb 20S OG proteasomes Cyclocarbonylates the active site Thr >1,000-fold more effective against Mtb than human proteasomes
Fellutamide B (Lin et al., 2010)	Lipopeptide aldehyde			Pre-clinical	β5	Inhibits wild type (Mtb20SWT) and open-gate mutant (Mtb20SOG) Mtb proteasomes
						Inhibits Mtb proteasome through one-step mechanism, inhibits hu20S proteasome through two-step mechanism
Syringolin (Totaro et al., 2017)	Natural products			Pre-clinical	β5	Species selective, bioactive Mtb inhibitors
Analogues A/B	Macrocyclic lactam attached to an exocyclic dipeptide urea					74-fold > selectivity for Mtb > hu20S proteasomes
						Cell-permeable, covalent, irreversible
DPLG2 (Lin et al., 2013)	Phenylimidazole-based,			Pre-clinical	β5	4,667-fold selective for Mtb proteasomes over human c20S and 3,647-fold over i20S proteasomes
	*N, C*-Capped dipeptide					Cell-permeable, kill non-replicating Mtb under nitrosative stress
B6 (Zhan et al., 2019)	Phenylimidazole peptidomimetic			Pre-clinical	β5	>12,500-fold selective for Mtb proteasomes than human c20S and i20S proteasomes
TDI5575 (Zhang and Lin, 2021)	Macrocyclic			Translational	β5ic>β5c>	Kills non-replicating Mtb under NO stress
	Peptides				β2i, β2c, β1i, β1c	Induces accumulation of pupylated proteins
						Stable in plasma
**MALARIA**						
Artemisinins (Bridgford et al., 2018)	Natural bioactive sesquiterpene lactone containing an unusual endoperoxide 1,2,4-trioxane ring			First-line treatment *for uncomplicated* *P. falciparum* malaria		Upregulation of the UPR Inhibits proteasome function Fast-acting against intraerythrocytic asexual blood-stage malaria Short half-life *in vivo*
Artesunate (Dondorp et al., 2010)	Semi-synthetic Lactol derivative			FDA-approved (IV) for severe malaria in adults and children		
Artemether (Esu et al., 2019)	Semi-synthetic Lactol derivative				β5>β1>>β2	Multi-pronged assault on protein homeostasis Activates ER stress, toxic accumulation of poly-Ub- proteins, kills parasites
WLW-vs (Stokes et al., 2019)	Peptide vinyl sulfones Irreversible			Pre-clinical	β2-selective	Plasmodium-selective, attenuate parasite growth *in vivo* in murine models
TDI-8304 (Zhan et al., 2021)	Macrocyclic peptide			Pre-clinical	β5	Species-selective Reduces parasitemia in humanized *P. falciparum*- infected mouse
PW28 (Tschan et al., 2013)	Peptido sulfonyl fluoride			Pre-clinical		Malaria
Carmaphycin B	(Cbz-LLLL-VF) Natural product					
analog 18 (LaMonte et al., 2017)	N-hexanoyl tripeptide, α,β-epoxyketone			Pre-clinical	β5	Inhibit β5 activity, blood-stage and gametocidal antimalarial activity
OZ439 (Charman et al., 2011)	Synthetic ozonide			Phase 2 trial		Good safety profile that clears parasitemia rapidly in both P. falciparum and P. vivax malaria
(Artefenomel)	Artemisinin pharmacophore					
MPI-5 (Xie et al., 2021)	Amino-amide boronate			Pre-clinical		Selective, potent anti-malarial activity across the parasite lifecycle, fast-acting, species selective over human proteasome, high selectivity for Pf cultures
						Oral availability, efficacy in SCID mouse model
MPI-13 (Xie et al., 2021)	Amino-amide boronate			Pre-clinical		Selective, potent anti-malarial activity across the parasite lifecycle, fast-acting, species selective over human proteasome, high selectivity for Pf cultures
						Oral availability, efficacy in SCID mouse model
**PROTOZOA**						
GNF5343 (Khare et al., 2016)	Azabenzoxazole			Investigational	β5	Inhibits kinetoplastid proteasome and growth
						Potent anti-*L. donovani* and anti-*T. brucei activity for the treatment of Leishmania, Chagas disease, and Sleeping sickness*
GNF6702 (101)	Azabenzoxazole			Investigational	β5	Optimization of GNF5343. Reduced risk of toxicity, improved selectivity over mammalian cell growth inhibition, low clearance, acceptable bioavailability and a 400-fold increase in potency against intra-macrophage *L. donovani* compared with GNF5343

Second-generation proteasome inhibitors (PIs) were developed to overcome bortezomib resistance as well as improved efficacy and safety and convenient methods of administration (Kisselev et al., 2012; Fricker, 2020). Carfilzomib (Onyx/Amgen) is derived from epoxomicin, – a tetrapeptide epoxyketone natural product isolated from *Actinomyces (* Kuhn et al., 2007; Huber and Groll, 2012; Kisselev et al., 2012; Herndon et al., 2013; Park et al., 2018; Fricker, 2020; Jayaweera et al., 2021). Carfilzomib is FDA-approved as a single agent for treatment of refractory MM, specifically those who had received >2 prior lines of therapy and have progressed on or within 60 days of completion of their last therapy (Kuhn et al., 2007; Herndon et al., 2013; Park et al., 2018). The oral PI ixazomib (Millennium-Takeda) is a peptide boronic acid, approved in combination with lenalidomide and dexamethasone, for MM patients who have received >1 prior therapy (Kumar et al., 2014; Moreau et al., 2016; Al-Salama et al., 2017). In adults with relapsed and/or refractory (RR)MM who previously had received 1-3 therapies, progression-free survival (PFS) was significantly prolonged in those who received ixazomib. A significantly longer time to progression and favorable hazard ratios for PFS were seen across all subgroups, including those with high risk cytogenetics.

## Proteasomes in Autoinflammatory Syndromes and Neurodevelopmental Disorders

Proteasomes are conserved throughout evolution dating back to *Archaea* because of the fundamental role in maintaining protein homeostasis. It therefore follows that proteasome mutations, while clinically rare, when detected would be associated with profound morbidity. Indeed, the proteasome-associated autoinflammatory syndromes (PRAAS) represent a spectrum of inherited autoinflammatory conditions first described in 1939 with characteristic skin findings, recurrent fevers, and joint contractures (Agarwal et al., 2010; Kitamura et al., 2011; Brehm and Kruger, 2015; Brehm et al., 2016). Related syndromes have been subsequently described, including Chronic atypical neutrophilic dermatoses with lipodystrophy and elevated temperature (CANDLE) syndrome, Nakajo-Nishimura syndrome, and Japanese autoinflammatory syndrome with lipodystrophy (McDermott et al., 2015). Mutations in the proteasome-immunoproteasome system drive the pathophysiology of these syndromes. Several groups independently described homozygous loss of function mutations in *PSMB8* that inhibit incorporation of the β5i into the 20S proteasome. The lack of *PSMB8* lead to ineffective response to IFN-γ induced oxidative stress, promoting sustained activation of IFN-γ and MAPK signaling driving chronic inflammation. Additional proteasome mutations have been linked to these syndromes including mutations in PSMG2/PAC2-Proteosome assembly chaperones (De Jesus et al., 2019). Management is challenging, involving chronic immune suppression with steroids and Janus Kinase inhibitors with allograft stem cell transplantation providing more durable clinical response (Sanchez et al., 2018; De Jesus et al., 2019; Verhoeven et al., 2022). These syndromes provide clinical evidence of the profound importance of proteasome function in cellular function and regulation of inflammatory responses.

The intracellular and extracellular accumulation of aggregated protein are linked to many diseases, including a number of proteinopathies, ageing-related neurodegeneration and systemic amyloidosis (Kopito, 2000; Olzmann et al., 2008). Cells avoid accumulating potentially toxic aggregates by mechanisms including the suppression of aggregate formation by molecular chaperones and the degradation of misfolded proteins by proteasomes. Importantly, proteasome inhibition drives formation of intracellular inclusions known as the aggresome, protein aggregates that tend to be refractory to proteolysis and to accumulate in inclusion bodies (Kawaguchi et al., 2003). Aggresome formation is a general response of cells which occurs when the capacity of the proteasome is exceeded by the production of aggregation-prone misfolded proteins. Aggresomes represents an important crossroads of the UPS and the unfolded protein response and are thought to be sequestered into autophagosomes before they are cleared by the lysosome. Large protein aggregates are unable to pass through the 20S proteasome, and increased protein aggregates can further proteasome inhibition, promoting a vicious cycle in neurodegenerative diseases, however proteasomes have been shown to co-localize with aggresomes. Hao and colleagues showed that Poh1, a proteasomal deubiquitinating enzyme, generates free K63-linked Ub chains that promote histone deacetylase 6 (HDAC6)-mediated aggresome clearance (Hao et al., 2013). HDAC6 has become a target for drug development to treat myeloma due to its major contribution in oncogenic cell transformation and overcomes resistance to PIs (Hideshima et al., 2016).

### Proteasome Inhibitors to Treat Multiple Myeloma

MM is described by expansion of clonal plasma cells that reside within bone marrow (BM), monoclonal protein detected in blood and/or urine, and end-organ dysfunction (Hideshima et al., 2001; Hideshima et al., 2007; Palumbo and Anderson, 2011; Munshi et al., 2017; Rajkumar, 2020; van de Donk et al., 2021). MM accounts for approximately 13% of all hematologic malignancies and is the second most common blood cancer in high-income and Western countries (Hideshima et al., 2007). The annual age-adjusted incidence is ~7.1 cases/100,000 persons in the U.S. with 34,920 new cases in 2021 and ~0.8% of all adults diagnosed with MM during their lifetime. Hideshima et al. demonstrated that bortezomib directly blocks myeloma proliferation, promotes killing of MM cell lines and freshly isolated patient MM cells and overcomes interleukin (IL)-6-mediated drug resistance (Moscvin et al., 2021). Bortezomib also reduces MM growth by impairing adherence to BMSCs and reducing their NF-κB-dependent upregulation of IL-6 secretion. Bortezomib disrupts paracrine signaling which reduces the proliferation and growth signaling of residual adherent MM cells. These results demonstrated that bortezomib directly acts on MM cells and disrupts BMSC-dependent pro-tumorigenic events within the BM milieu (Kumar and Rajkumar, 2008; Moscvin et al., 2021).

Perturbations in intracellular protein homeostasis including the accumulation of ubiquitinated proteins is accompanied by a type I interferon (IFN) signature (Ebstein et al. 2019) Under sterile conditions, the unfolded protein response (UPR), typically initiated in response to an accumulation of unfolded and/or misfolded proteins in the endoplasmic reticulum (ER), i.e., ER stress response, triggers type I IFN. Since PRAAS are caused by inherited and/or *de novo* loss-of-function mutations in genes encoding proteasome subunits, e.g., *PSMB8, PSMB9, PSMB7, PSMA3*, or proteasome assembly factors including *POMP* and *PSMG2*, respectively (Poli et al., 2018; De Jesus et al., 2019). The recent observation that the UPR is engaged in subjects carrying *POMP* mutations strongly suggests a possible implication in the cause-and-effect relationship between proteasome impairment and interferonopathy onset. Proteasome inhibition is similarly associated with an induction of ER stress. Studencka-Turski et al. proposed a model where proteasome inhibition inhibits subsequent retro-translocation of misfolded ER proteins (Studencka-Turski et al., 2019). The buildup of these misfolded proteins activates all three cellular sensors of ER stress including Activation transcription factor 6 (ATF6), protein kinase R-like ER kinase and inositol-requiring protein 1-α (IRE-1α). They subsequently link IRE-1α activity to the type I IFN response. The ERAD pathway is primarily defined as an ER-localized UPS that ensures the proteasome-mediated degradation of misfolded proteins trafficking in the ER (Liu and Kaufman, 2003; Schroder and Kaufman, 2005; Hetz et al., 2015). Damaged proteins and/or proteins aberrantly modified are transported from the ER lumen back to the cytosol by retro-translocation prior to subsequent ubiquitination and membrane extraction for degradation by proteasomes. Nearly ~30% of all proteins are synthesized at the ER by the secretory pathway and, as such, potential ERAD substrates make ERAD a reliable sensor to alarm cells to decreased ability to degrade proteins. The type-I IFN response also plays a key role in the efficacy of bortezomib in MM therapy. Gulla and colleagues demonstrated that bortezomib treatment modulates the immunosuppressive microenvironment in MM patients, promoting type-I IFN signaling through a cGAS/STING-mediated pathway (Gulla et al., 2021).

The immunoproteasome is classically associated with presentation of antigenic peptides presented by MHC class I molecules due to its higher cleavage activity after hydrophobic residues, but this hydrophobic processivity has important implications for antigen repertoire. Indeed, Chapiro et al. addressed this issue by analyzing processing of three clinically relevant antigens, showing that the gp100_208-217_ and peptide tyrosinease_369-377_ antigens are destroyed secondary to cleavage after an internal hydrophobic residue, while the peptide MAGE-C2_336-344_ is destroyed by the constitutive proteasome due to cleavage after an acidic residue (Chapiro et al., 2006). Thus, the balance of constitutive and immunoproteasomes together play a key role in the antigen repertoire.

### Proteasome Inhibitors to Treat Mantle Cell Lymphoma

Mantle cell lymphoma (MCL) is a subtype of Non-Hodgkin’s Lymphoma (NHL) which has a relatively poor prognosis, despite numerous cytotoxic chemotherapy options (Cheah et al., 2016). Bortezomib, initially approved for patients with RRMM, is also approved for RR MCL (Holkova and Grant, 2012). Robak et al. investigated whether switching bortezomib for vincristine in frontline therapy with R-CHOP (rituximab, cyclophosphamide, doxorubicin, vincristine, and prednisone) improved outcome in newly diagnosed ND MCL patients (Robak et al., 2015). Bortezomib, rituximab, cyclophosphamide, doxorubicin and prednisone was more effective than R-CHOP for ND MCL but at the cost of increased hematologic toxicity.

### Proteasome Inhibitors to Treat Diffuse Large B Cell Lymphoma

While the survival rate of DLBCL has significantly improved, many patients do not achieve CR or relapse, especially those diagnosed with the activated B cell-like (ABC) subtype. Bortezomib exhibits activity in DLBCL, especially the ABC DLBCL. A meta-analysis compared the efficacy and adverse events in bortezomib-containing regimens with standard R-CHOP treatment. Compared to R-CHOP, the bortezomib-containing regimen did not prolong survival of ABC DLBCL patients and those who received bortezomib had greater risk of peripheral neuropathy (Dunleavy et al., 2009; Lin et al., 2018).

HIV+ patients exhibit much higher risk of developing DLBCL than an HIV- patient. These lymphomas are often associated with Epstein Barr Virus (EBV) and Human Herpes Virus 8 (HHV8) infection. Reid et al. postulated adding bortezomib to rituximab, ifosfamide, carboplatin and etoposide salvage therapy could activate latent viral infections and improve lymphoma control. Their regimen exhibits a 77% response rate and 57% 1-year survival, outperforming historic controls associated with increased viral titers of EBV and HIV was undetectable throughout the study. This regimen is recommended *per* NCCN guidelines for RR DLBLC in HIV+ patients with grade 2B evidence (Reid et al., 2018).

### Proteasome Inhibitors to Treat Relapsed/Refractory Idiopathic Multicentric Castleman’s

Treatment of RR idiopathic Multicentric Castleman’s disease (RR iMCD) remains challenging and often has a poor prognosis (Cabot et al., 1954). Zhang et al. evaluated a bortezomib-cyclophosphamide-dexamethasone (BCD) as a regimen in 24 RR iMCD patients (Zhang et al., 2020). The estimated 1-year PFS and OS were 79% and 92%, respectively, and BCD was a safe, effective option in RR iMCD.

## Targeting Proteasomes to Treat Infectious Disease

*Archaea* are single celled organisms now recognized as a third domain of life (Eme et al., 2017). Molecular and genetic evidence suggests that *Eukarya* are related to *Archaea* and indicate that eukaryotes descend from *Archaea (* Eme et al., 2017). Like eukaryotes, *Archaea* are thought to use the proteasome as a primary system for energy-dependent protein degradation (Löwe et al., 2015; Eme et al., 2017; Majumder et al., 2019). Proteasomes from *Archaea* are highly related, but distinct, from eukaryotic forms in their structural organization and functional activities. While proteasomes perform critical functions in bacteria, they are not as absolutely essential as in *Archaea* and eukaryotes. Taken together, accumulating evidence indicate that proteasomal inhibition can affect the pathogenicity of select microbial pathogens. Therefore, it is reasonable that pharmacologic inhibition of proteasomes in organisms less evolutionarily developed than humans may represent a novel therapeutic approach to improve the treatment of infectious diseases caused by protozoans and bacteria.

### Proteasome Inhibitors to Treat Malaria

Malaria ranks as one of the greatest global health problems and each year *P. falciparum* malaria infects >200 million individuals and causes >400,000 deaths (Phillips et al., 2017; World Health Organization, 2020). *Plasmodium* parasites, which are spread to humans through the bites of infected female *Anopheles* mosquitoes, causes the acute, febrile illness malaria. At least 5 species cause malaria in humans. *P. falciparum*, most prevalent in Africa and *P. vivax*, dominant outside of sub-Saharan Africa, pose the greatest threat to humans (Phillips et al., 2017; World Health Organization, 2020; Bergmann et al., 2021). Current antimalarial control relies on artemisinin-based combination therapies (ACTs) which have a cure failure rate of ∼50% in portions of Southeast Asia (Van de Pluijim et al., 2019). Zani et al., 2014 Identification of novel actionable targets, active against all parasite life cycle stages and that prevent and/or overcome drug resistance remains an unmet need.

The *Plasmodium* proteasome represents a novel, actionable, therapeutic target to improve malaria treatment and disease prevention (https://www.who.int/publications/digital/global-tuberculosis-report-2021; Li et al., 2012; Li et al., 2014; Dogovski et al., 2015; Li et al., 2016; Ng et al., 2017; Kirkman et al., 2018; Krishnan and Williamson, 2018; Xie et al., 2018; Yoo et al., 2018; Van de Pluijim et al., 2019; Bergmann et al., 2021; Zhan et al., 2021). The proteasome is essential throughout the *Plasmodium* life cycle as parasites quickly adapt to a new host and undergo morphologic changes during asexual replication and sexual differentiation. Because of the high replication rate during the erythrocytic stage of parasites, protein quality control is critical for *P. falciparum* survival. Inhibiting the *P. falciparum* proteasome represents a drug target that may improve current treatment and avoid overcome ACT-resistance. *Plasmodium* harbor 3 types of protease complexes: a eukaryotic (26S) cytoplasmic and nuclear proteasome, a prokaryotic mitochondrial proteasome homologous to ClpQ, and a caseinolytic, apicoplast ClpP protease (Krishnan and Williamson, 2018).

Structural information obtained for the *P. falciparum* proteasomal complex renewed efforts to generate and validate new PIs as well as to re-examine the previously studied PI classes, namely; dipeptidyl boronic acids, α,β-epoxyketones, β-lactones, peptide aldehydes, vinyl sulfones and cyclic peptides (**Table 1**). Compounds representative of each PI class were evaluated using proteasomes from *Plasmodium (* Li et al., 2012; Li et al., 2014; Dogovski et al., 2015; Li et al., 2016; Ng et al., 2017; Kirkman et al., 2018; Krishnan and Williamson, 2018; Xie et al., 2018; Yoo et al., 2018; Van de Pluijim et al., 2019; Zhan et al., 2021). Li et al. screened a library of 670 carfilzomib-based analogs to detect those that selectively cytotoxic to parasites (Li et al., 2012). The authors identified PR3, which *in vitro* significantly killed the parasite but displayed dramatically reduced toxicity against the host. Parasite-specific toxicity was not due to selective targeting of *Plasmodium* proteasomes over host proteasomes, but rather, attributed to lack of efficacy against a human proteasome subunit. PR3 was later shown to significantly reduce the parasite load in *P. berghei*-infected mice in the absence of host toxicity. The results validated the proteasome as a viable anti-malarial drug target. Li et al. then employed active site specific probes to explore the *Plasmodium* proteasome. They demonstrated that PIs often do not exhibit the same subunit-targeting profiles for host and parasite proteasomes (Li et al., 2014). *Plasmodium* parasites undergoing schizogony, a biologic phenomenon not present in human cell types, are highly sensitive to selective inhibition of proteasomal Ch-T-like activity. However, not all stages of the parasite are equally PI-sensitive. Most significant parasite killing results were obtained from co-inhibition of multiple *Plasmodium* proteasome catalytic subunits.

Dogovski et al. performed a kinetic analysis to compare drug responses of K13 wild-type and mutant isolates of *P. falciparum*, sourced from the Cambodian Pailin province (Dogovski et al., 2015). The authors determined that artemisinin slows parasite proliferation and induces Ub-protein conjugate levels. Interestingly, drug-resistant parasites exhibited a lower amount of Ub-conjugates as well as delayed onset of death. Carfilzomib strongly synergized with artemisinin activity against both sensitive and resistant parasites and synergy was observed against *P. berghei in vivo*. Kirkman et al. identified inhibitors with improved selectivity for the malarial proteasome β5 subunit over each active subunit of human proteasomes (Kirkman et al., 2018). The agents killed *Plasmodium* at all stage of the life cycle and interacted synergistically with both artemisinin and a β2 subunit inhibitor. Yoo et al. found that an optimized electrophilic warhead was needed to enable high selectivity and that toxicity was dictated by the extent of co-inhibition of human β2 and β5 catalytic subunits (Kaiser et al., 2007). The authors identified compounds with >3 orders of magnitude selectivity for *Plasmodium* proteasomes that were optimized for high potency, selectivity, solubility, metabolic stability and oral bioavailability (Yoo et al., 2018). Li et al. employed a substrate profiling technique to detect specificity differences between *P. falciparum* and human proteasomes. They designed inhibitory molecules based upon amino-acid preferences specific to the parasite proteasome, and found that these compounds preferentially inhibited the β2-subunit (Li et al., 2016). They employed cryo-electron microscopy and single-particle analysis at a resolution of 3.6 Å to determine the structure of the inhibitor bound-*P. falciparum* 20S proteasome. Results revealed an unusually open *P. falciparum* β2 active site and revealed information to describe the architecture of the active-site as well as information to better refine inhibitor design. Xie et al. characterized potent, specific, amino-amide boronates that inhibited the *P. falciparum* 20S proteasome (Pf20S) β5 active site and demonstrated rapid antimalarial activity (Xie et al., 2018). These boronates selectively impaired *P. falciparum* growth compared to human cells and showed significant potency against *P. falciparum* and *P. vivax* field isolates. The compounds also displayed oral efficacy in a murine model of *P. falciparum* malaria. Zhan et al. developed a noncovalent, macrocyclic peptide inhibitor of the malarial proteasome with high species selectivity and favorable pharmacokinetics. The compound specifically inhibited the *Pf20S* β5 catalytic subunit, killed artemisinin-sensitive and -resistant *P. falciparum* isolates *in vitro* and reduced parasitemia in a murine model of *P. falciparum (* Zhan et al., 2021).

As reported by Ng et al., recent advances have lead agents that specifically target the *Plasmodium* proteasome to demonstrate that targeting the UPP and other non-proteasomal protein clearance pathways to disrupt proteostasis is an attractive avenue to combat drug resistance (Ng et al., 2017).

### Proteasome Inhibitors to Treat Bacterial Infections

*Mycobacterium tuberculosis* (Mtb), a highly lethal bacteria, is the focus of numerous active pharmaceutical development programs. The estimated incidence of TB suggests that ~10 million people fell ill with disease in 2020 (https://www.who.int/publications/digital/global-tuberculosis-report-2021). The global number of TB deaths increased between 2019 and 2020 from ~1.2 million to 1.3 million among HIV-negative people and from 209,000 to 214,000 among the HIV-positive (https://www.who.int/publications/digital/global-tuberculosis-report-2021). While the disease can be managed, many individuals develop drug resistance after attempting the first line of drug therapy- rifampicin, pyrazinamide, isoniazid, and ethambutol (https://www.who.int/publications/digital/global-tuberculosis-report-2021). Unfortunately, many TB patients do not have access to the care needed to maintain these treatments and two forms of drug resistant TB exist, multi-drug resistant TB described as resistance to rifampin and isoniazid, and extensive drug resistant TB described as disease refractory to rifampin and isoniazid, any fluoroquinolone, and one or more second-line drug.

*Mtb* require a functioning proteasome to elicit disease and encode a proteasome that is necessary for fatal effects in mice. Lin et al. sought to develop *Mtb*-selective PIs and identified oxathiazol-2-one compounds (Lin et al., 2009). These agents are cytotoxic to non-replicating Mtb and act as suicide-substrate inhibitors of *Mtb* proteasomes by cyclocarbonylating the active site Thr. A high-throughput, natural compound library screen detected fellutamide B, a lipopeptide aldehyde, as a potent inhibitor of *Mtb* proteasomal activity (Lin et al., 2010). An *in vitro* screen of ~20,000 compounds detected 2 oxathiazol-2-one compounds, GL5 and HT1171, which effectively inhibit *Mtb* proteasomes *(* Totaro et al., 2017). Using a model of nitroxidative stress that induces a pathologically relevant state of dormancy, GL5 and HT1171 also generated concentration-dependent *Mtb* death, whereas there was no apparent cell death in mammalian cell lines at comparable or higher concentrations. Kinetics experiments showed that GL5 and HT1171 were >1,000-fold more effective against *Mtb* proteasomes than human proteasomes to support species-specific selectivity. Using a molecular docking and simulation approach, Tyagi et al. screened a small molecule library from Medicines for Malaria Venture against the proteasome (Tyagi et al., 2022). They identified MMV019838 and MMV687146 which actively interacted with the catalytic domain of *Mtb* proteasomes and inhibited *Mtb* growth *in vitro*. Furthermore, their studies demonstrated strong and stable interaction of these molecules with *Mtb* proteasomes compared to that observed with human proteasomes.

### Proteasome Inhibitors to Treat Other Parasitic Infections

Proteasomes have been isolated and studied in *Giardia, Entamoeba, Leishmania, Trypanosoma, Plasmodium* and *Toxoplasma* spp.; all protozoan parasites of medical importance (Robertson, 1999; Shaw et al., 2000; De Diego et al., 2001; Makioka et al., 2002; Paugam et al., 2003; Khare et al., 2016). Lactacystin demonstrates wide-ranging activity *in vitro* blocking the growth of *Entamoeba histolytica* and *E. invadens (* Makioka et al., 2002; Paugam et al., 2003). In *L. mexicana*, the proteasome regulates the cell cycle and is essential for growth (Robertson, 1999). Surprisingly, lactacystin did not inhibit the *Leishmania* proteasome *in situ*. The proteasome has also been explored in the causative agent of Chagas disease, *Trypanosoma cruzi (* De Diego et al., 2001). GNF6702 is a selective inhibitor of the kinetoplastid proteasome, demonstrated unprecedented *in vivo* efficacy, cleared parasites from mice in 3 infection models and was well-tolerated (Khare et al., 2016). Importantly, GNF6702 did not inhibit the mammalian proteasome (Khare et al., 2016). Proteasomes are also active within the intracellular pathogen *Toxoplasma* during development and replication, representing another setting for PI therapy (Shaw et al., 2000).

## Conclusions and Perspectives

Features of the UPP are conserved throughout evolution from *Archaea* to humans. The proteasome represents an essential component of cellular biology and is a clinically validated, actionable target that can be pharmacologically modulated to treat human diseases. FDA-approved PIs have significantly improve the quality-of-life and overall survival of those afflicted with MM. Plasma cells are antibody-producing factories that synthesize and secrete vast amounts of immunoglobulins, and hence, are highly dependent on mechanisms that maintain proteostasis. Pharmacologic blockade of the UPP highlights the hypersensitivity of MM cells to agents that disrupt the delicate balance of protein synthesis and degradation. Similarly, that same rationale has been applied for the off-label use of proteasome and immunoproteasome inhibitors to target plasma cells that contribute to antibody-mediated rejection that precludes organ transplantation and autoimmune diseases (Johnson et al., 2017; Woodle et al., 2017; Schmidt et al., 2018; Xi et al., 2019; Woodle et al., 2020). KZR-616 is a first-in-class selective inhibitor of the immunoproteasome, which is active in >15 autoimmune disease models, including murine models of systemic lupus erythematosus (SLE) and lupus nephritis (LN) (Muchamuel et al., 2018; Xi et al., 2019). Selective inhibition of the immunoproteasome modulates both innate and adaptive immune effector cells, resulting in reduced inflammatory T helper cell subsets (Th1 and Th17), increased T regs, and decreased plasma cells and autoantibodies.

More effective drugs are urgently needed to treat parasitic diseases that persist as global health threats and leading causes of death, especially in the developing world. Proteasomes contained within pathogenic organisms possess a structural architecture and Ch-T-like active sites similar to that described in eukaryotic forms that are readily accessible and sensitive to pharmacologic inhibition. However, agents intended to target proteasomes in pathogenic microbes must spare human proteasomes to prevent unwanted toxicity and immunosuppression. Moreover, drug resistance inevitably emerges during the course of treatment in MM patients through a multitude of molecular mechanisms (Driscoll and Brailey, 2017; Bo Kim, 2021). PIs that target malaria and Tb may potentially have a greater value for the treatment of patients that have developed resistance to current, standard-of-care agents. Since malaria and Tb affect individuals in the developing world, often children and those of child-bearing age, PIs to treat these diseases need to be not only orally bioavailable, cost-effective and curative following a short treatment course but also safe and non-teratogenic as well. As witnessed by advances over the past two decades in oncology, PIs offer potential as transformative agents in the treatment of infectious diseases in the upfront setting or when standard-of-care agents are ineffective and drug-resistant disease has emerged.

## Author Contributions

JD, JI-H and EM developed the concept for the article, prepared the figure and table, wrote and edited the review. Each author made a substantial contribution to the manuscript and approved the final version. All authors contributed to the article and approved the submitted version.

## Funding

Research was supported by NIH R01 (5R01AI139141 to JD), University Hospitals Cleveland Medical Center/Seidman Cancer Center, and the Case Comprehensive Cancer Center.

## Conflict of Interest

The authors declare that the research was conducted in the absence of any commercial or financial relationships that could be construed as a potential conflict of interest.

## Publisher’s Note

All claims expressed in this article are solely those of the authors and do not necessarily represent those of their affiliated organizations, or those of the publisher, the editors and the reviewers. Any product that may be evaluated in this article, or claim that may be made by its manufacturer, is not guaranteed or endorsed by the publisher.

## References

[B1] Agarwal A. K. Xing C. DeMartino G. N. Mizrachi D. Hernandez M. D. Sousa A. B.. (2010). *PSMB8* Encoding the β5i Proteasome Subunit is Mutated in Joint Contractures, Muscle Atrophy, Microcytic Anemia, and Panniculitis-Induced Lipodystrophy Syndrome. Am. J. Hum. Genet. 87(6):866–872. doi: 10.1016/j.ajhg.2010.10.031 21129723 PMC2997366

[B2] Al-Salama Z. T. Garnock-Jones K. P. Scott L. J. (2017). Ixazomib: A Review in Relapsed and/or Refractory Multiple Myeloma. Target Oncol. 12 (4), 535–542. doi: 10.1007/s11523-017-0504-7 28660423

[B3] Available at: https://www.who.int/publications/digital/global-tuberculosis-report-2021.

[B4] Bergmann C. van Loon W. Habarugira F. Tacoli C. Jäger J. C. Savelsberg D.. (2021). Increase in Kelch 13 Polymorphisms in Plasmodium Falciparum, Southern Rwanda. Emerg. Infect. Dis. 27(1), 294–296. doi: 10.3201/eid2701.203527 33350925 PMC7774571

[B5] Bo Kim K. (2021). Proteasomal Adaptations to FDA-Approved Proteasome Inhibitors: A Potential Mechanism for Drug Resistance? Cancer Drug Resist. 4, 634–645. 34308274 10.20517/cdr.2021.27 PMC8297691

[B6] Brehm A. Kruger E. (2015). Dysfunction in Protein Clearance by the Proteasome: Impact on Autoinflammatory Diseases. Semin. Immunopathol. 37 (4), 323–333. doi: 10.1007/s00281-015-0486-4 25963519

[B7] Brehm A. Liu Y. Sheikh A. Marrero B. Omoyinmi E. Zhou Q.. (2016). Additive Loss-of-Function Proteasome Subunit Mutations in CANDLE/PRAAS Patients Promote Type I IFN Production. J. Clin. Invest 1126(11), 4196–211. doi: 10.1172/JCI86020 PMC4639987 26524591

[B8] Bridgford J. L. Xie S. C. Cobbold S. A. Pasaje C. F. A. Herrmann S. Yang T.. (2018). Artemisinin Kills Malaria Parasites by Damaging Proteins and Inhibiting the Proteasome. Nat. Commun. 9(1), 3801. doi: 10.1038/s41467-018-06221-1 30228310 PMC6143634

[B9] Brown M. Driscoll J. J. Monaco J. (1991). Structural and Serological Similarity of MHC-Linked LMP and Proteasome (Multicatalytic Proteinase) Complexes. Nature 353, 355–357. doi: 10.1038/353355a0 1922341

[B10] Cabot R. C. Castleman B. Towne V. W. (1954). CASE Records of the Massachusetts General Hospital Weekly Clinicopathological Exercises: Case 40011. N Engl. J. Med. 250, 26–30. doi: 10.1056/NEJM195401072500107 13194083

[B11] Chapiro J. Claverol S. Piette F. Ma W. Stroobant V. Guillaume B.. (2006). Destructive Cleavage of Antigenic Peptides Either by the Immunoproteasome or by the Standard Proteasome Results in Differential Antigen Presentation. J. Immunol. 176, 1053–1061. doi: 10.4049/jimmunol.176.2.1053 16393993

[B12] Charman S. A. Arbe-Barnes S. Bathurst I. C. Brun R. Campbell M. Charman W. N.. (2011). Synthetic Ozonide Drug Candidate OZ439 Offers New Hope for a Single-Dose Cure of Uncomplicated Malaria. Proc. Natl. Acad. Sci. U.S.A. 108, 4400–4405. doi: 10.1073/pnas.1015762108 21300861 PMC3060245

[B13] Cheah C. Y. Seymour J. F. Wang M. L. (2016). Mantle Cell Lymphoma. J. Clin. Oncol. 34, 1256–1269. doi: 10.1200/JCO.2015.63.5904 26755518

[B14] Ciechanover A. (2005). Proteolysis: From the Lysosome to Ubiquitin and the Proteasome. Nat. Rev. Mol. Cell Biol. 6 (1), 79–87. doi: 10.1038/nrm1552 15688069

[B15] Cromm P. M. Crews C. M. (2017). The Proteasome in Modern Drug Discovery: Second Life of a Highly Valuable Drug Target. ACS Cent Sci. 3, 830–8 8. doi: 10.1021/acscentsci.7b00252 28852696 PMC5571462

[B16] Dahlmann B. Kopp F. Kuehn L. Niedel B. Pfeifer G. Hegerl R. Baumeister W.. (1989). The Multicatalytic Proteinase (Prosome) is Ubiquitous From Eukaryotes to Archaebacteria. FEBS Lett. 251(1-2), 125–131. doi: 10.1016/0014-5793(89)81441-3 2502434

[B17] Dahlmann B. Kuehn L. Reinauer H. (1983). FEBS Lett. 160, 243–247. 6350043 10.1016/0014-5793(83)80975-2

[B18] De Diego J. L. Katz J. M. Marshall P.. (2001). The Ubiquitin-Proteasome Pathway Plays an Essential Role in Proteolysis During Trypanosoma Cruzi Remodeling. Biochemistry 40 (4), 1053–1062. doi: 10.1021/bi001659k 11170428

[B19] De Jesus A. A. Brehm A. Vantries R. Pillet P. Parentelli A. S. Montealegre Sanchez G. A.. (2019). Novel Proteasome Assembly Chaperone Mutations in PSMG2/PAC2, Cause the Autoinflammatory Interferonopathy, CANDLE/Praas4. J. Allergy Clin. Immunol. 143, 1939–43.e8. doi: 10.1016/j.jaci.2018.12.1012 30664889 PMC6565382

[B20] Dick L. R. Fleming P. E. (2010). Building on Bortezomib: Second Generation Proteasome Inhibitors as Anti-Cancer Therapy. Drug Discovery Today 15, 243–249. doi: 10.1016/j.drudis.2010.01.008 20116451

[B21] Dick T. P. Nussbaum A. K. Deeg M. Heinemeyer W. Groll M. Schirle M.. (1998). Contribution of Proteasomal Beta-Subunits to the Cleavage of Peptide Substrates Analyzed With Yeast Mutants. J. Biol. Chem. 273 (40), 25637–25646. doi: 10.1074/jbc.273.40.25637 9748229

[B22] Dikic I. (2017). Proteasomal and Autophagic Degradation Systems. Annu. Rev. Biochem. 86, 193–224. doi: 10.1146/annurev-biochem-061516-044908 28460188

[B23] Dogovski C. Xie S. C. Burgio G. Bridgford J. Mok S. McCaw J. M.. (2015). Targeting the Cell Stress Response of Plasmodium Falciparum to Overcome Artemisinin Resistance. PloS Biol. 13(4), e1002132. doi: 10.1371/journal.pbio.1002132 25901609 PMC4406523

[B24] Dondorp A. M. Fanello C. I. Hendriksen I. C. Gomes E. Seni A. Chhaganlal K. D.. (2010). Artesunate Versus Quinine in the Treatment of Severe Falciparum Malaria in African Children (AQUAMAT): An Open-Label, Randomised Trial. Lancet 376(9753), 1647–1657. doi: 10.1016/S0140-6736(10)61924-1 21062666 PMC3033534

[B25] Driscoll J. J. Brailey M. (2017). Emerging Small Molecule Approaches to Enhance the Antimyeloma Benefit of Proteasome Inhibitors. Cancer Metastasis Rev. 36 (4), 585–598. doi: 10.1007/s10555-017-9698-5 29052093

[B26] Driscoll J. Brown M. Finley D. Monaco J. J.. (1993). MHC-Linked *LMP* Gene Products Specifically Alter Peptidase Activities of the Proteasome. Nature 365(6443), 262–264. doi: 10.1038/365262a0 8371781

[B27] Driscoll J. J. Finley D. (1992). A Controlled Breakdown: Antigen Processing and the Turnover of Viral Proteins. Cell 68, 823–825. doi: 10.1016/0092-8674(92)90024-7 1312390

[B28] Driscoll J. Goldberg A. L. (1989). Skeletal Muscle Proteasome can Degrade Proteins in an ATP-Dependent Process That Does Not Require Ubiquitin. Proc. Natl. Acad. Sci. U S A. 86, 787–791. doi: 10.1073/pnas.86.3.787 2536933 PMC286562

[B29] Driscoll J. Goldberg A. L. (1990). The Proteasome (Multicatalytic Protease) Is a Component of the 1500-kDa Proteolytic Complex Which Degrades Ubiquitin-Conjugated Proteins. J. Biol. Chem. 265, 4789–4792. doi: 10.1016/S0021-9258(19)34041-4 2180950

[B30] Dunleavy K. Pittaluga S. Czuczman M. S.. (2009). Differential Efficacy of Bortezomib Plus Chemotherapy Within Molecular Subtypes of Diffuse Large B-Cell Lymphoma. Blood 113 (24), 6069–6076. doi: 10.1182/blood-2009-01-199679 19380866 PMC2699229

[B31] Ebstein F. Poli Harlowe M. C. Studencka-Turski M. Krüger E. (2019). Contribution of the Unfolded Protein Response (UPR) to the Pathogenesis of Proteasome-Associated Autoinflammatory Syndromes (PRAAS). Front. Immunol. 10. doi: 10.3389/fimmu.2019.02756 PMC6890838 31827472

[B32] Eme L. Spang A. Lombard J. Stairs C. W. Ettema T. J. G.. (2017). Archaea Origin Eukaryotes. Nat. Rev. Microbiol. 15, 711–723. doi: 10.1038/nrmicro.2017.133 29123225

[B33] Esu E. B. Effa E. E. Opie O. N. Meremikwu M. M. (2019). Artemether for Severe Malaria. Cochrane Database Syst. Rev. 6 (6), CD010678. doi: 10.1002/14651858.CD010678.pub3 31210357 PMC6580442

[B34] Eytan E. Ganoth D. Armon T. Hershko A.. (1989). ATP-Dependent Incorporation of 20S Protease Into the 26S Complex That Degrades Proteins Conjugated to Ubiquitin. Proc. Natl. Acad. Sci. U.S.A. 86(20), 7751–7755. doi: 10.1073/pnas.86.20.7751 2554287 PMC298148

[B35] Falkenburg P. E. Haass C. Kloetzel P. M. Niedel B. Kopp F. Kuehn L.. (1988). Drosophila Small Cytoplasmic 19S Ribonucleoprotein is Homologous to the Rat Multicatalytic Proteinase. Nature 331(6152), 190–192. doi: 10.1038/331190a0 3123994

[B36] Finley D. (2009). Recognition and Processing of Ubiquitin-Protein Conjugates by the Proteasome. Annu. Rev. Biochem. 78, 477–513. doi: 10.1146/annurev.biochem.78.081507.101607 19489727 PMC3431160

[B37] Fricker L. D. (2020). Proteasome Inhibitor Drugs. Annu. Rev. Pharmacol. Toxicol. 6, 457–476. doi: 10.1146/annurev-pharmtox-010919-023603 31479618

[B38] Gallastegui N. Groll M. (2010). The 26S Proteasome: Assembly and Function of a Destructive Machine. Trends Biochem. Sci. 35 (11), 634–642. doi: 10.1016/j.tibs.2010.05.005 20541423

[B39] Groll M. Ditzel L. Löwe J. Stock D. Bochtler M. Bartunik H. D.. (1997). Structure of 20S Proteasome From Yeast at 2.4Å Resolution. Nature 386, 463–471. doi: 10.1038/386463a0 9087403

[B40] Gulla A. Morelli E. Samur M. K. Botta C. Hideshima T. Bianchi G.. (2021). Bortezomib Induces Anti–Multiple Myeloma Immune Response Mediated by cGAS/STING Pathway Activation. Blood Cancer Discovery 2, 468–483. doi: 10.1158/2643-3230.BCD-21-0047 34568832 PMC8462183

[B41] Hao R. Nanduri P. Rao Y. Panichelli R. S. Ito A. Yoshida M.. (2013). Proteasomes Activate Aggresome Disassembly and Clearance by Producing Unanchored Ubiquitin Chains. Mol. Cell. 51 (6), 819–828. doi: 10.1016/j.molcel.2013.08.016 24035499 PMC3791850

[B42] Herndon T. M. Deisseroth A. Kaminskas E. Kane R. C. Koti K. M. Rothmann M. D.. (2013). U.S. Food and Drug Administration Approval: Carfilzomib for the Treatment of Multiple Myeloma. Clin. Cancer Res. 19, 4559–4563. doi: 10.1158/1078-0432.CCR-13-0755 23775332

[B43] Hetz C. Chevet E. Oakes S. A. (2015). Proteostasis Control by the Unfolded Protein Response. Nat. Cell Biol. 17, 829–838. doi: 10.1038/ncb3184 26123108 PMC5546321

[B44] Hideshima T. Richardson P. Chauhan D. Palombella V. J. Elliott P. J. Adams J.. (2001). The Proteasome Inhibitor PS-341 Inhibits Growth, Induces Apoptosis, and Overcomes Drug Resistance in Human Multiple Myeloma Cells. Cancer Res. 61, 3071–3076. 11306489

[B45] Hideshima T. Mitsiades C. Tonon G. Richardson P. G. Anderson K. C.. (2007). Understanding Multiple Myeloma Pathogenesis in the Bone Marrow to Identify New Therapeutic Targets. Nat. Rev. Cancer 7, 585–598. doi: 10.1038/nrc2189 17646864

[B46] Hideshima T. Qi J. Paranal R. M. Tang W. Greenberg E. West N.. (2016). Discovery of Selective Small-Molecule HDAC6 Inhibitor for Overcoming Proteasome Inhibitor Resistance in Multiple Myeloma. Proc. Natl. Acad. Sci. U S A. 113 (46), 13162–13167. doi: 10.1073/pnas.1608067113 27799547 PMC5135369

[B47] Holkova B. Grant S. (2012). Proteasome Inhibitors in Mantle Cell Lymphoma. Best Pract. Res. Clin. Haematol. 25 (2), 133–141. doi: 10.1016/j.beha.2012.04.007 22687449 PMC3374152

[B48] Huber E. M. Groll M. (2012). Inhibitors for the Immuno- and Constitutive Proteasome: Current and Future Trends in Drug Development. Angew Chem Int. Ed 51, 8708–8720. doi: 10.1002/anie.201201616 22711561

[B49] Jayaweera S. P. E. Wanigasinghe Kanakanamge S. P. Rajalingam D.. (2021). Carfilzomib: A Promising Proteasome Inhibitor for the Treatment of Relapsed and Refractory Multiple Myeloma. Front. Oncol. 11, 740796. doi: 10.3389/fonc.2021.740796 34858819 PMC8631731

[B50] Johnson H. W. B. Anderl J. L. Bradley E. K. Bui J. Jones J. Arastu-Kapur S.. (2017). Discovery of Highly Selective Inhibitors of the Immunoproteasome Low Molecular Mass Polypeptide 2 (LMP2) Subunit. ACS Med. Chem. Lett. 8 (4), 413–417. doi: 10.1021/acsmedchemlett.6b00496 28435528 PMC5392757

[B51] Kaiser M. Wittlin S. Nehrbass-Stuedli A. Dong Y. Wang X. Hemphill A.. (2007). Peroxide Bond-Dependent Antiplasmodial Specificity of Artemisinin and OZ277 (Rbx11160). Antimicrob. Agents Chemother. 51(8), 2991–2993. doi: 10.1128/AAC.00225-07 17562801 PMC1932508

[B52] Kawaguchi Y. Kovacs J. J. McLaurin A. Vance J. M. Ito A. Yao T.-P. (2003). The Deacetylase HDAC6 Regulates Aggresome Formation and Cell Viability in Response to Misfolded Protein Stress. Cell 115 (6), 727–738. doi: 10.1016/S0092-8674(03)00939-5 14675537

[B53] Khare S. Nagle A. S. Biggart A. Lai Y. H. Liang F. Davis L. C.. (2016). Proteasome Inhibition for Treatment of Leishmaniasis, Chagas Disease and Sleeping Sickness. Nature 537 (7619), 229–233. doi: 10.1038/nature19339 27501246 PMC5161665

[B54] Kirkman A. Zhan W. Visone J. Dziedziech A. Singh P. K. Fan H.. (2018). Antimalarial Proteasome Inhibitor Reveals Collateral Sensitivity From Intersubunit Interactions and Fitness Cost of Resistance. Proc. Natl. Acad. Sci. U.S.A. 115(29), E6863–E6870. doi: 10.1073/pnas.1806109115 29967165 PMC6055138

[B55] Kisselev A. F. van der Linden W. A. Overkleeft H. S. (2012). Proteasome Inhibitors: An Expanding Army Attacking a Unique Target. Chem. Biol. 19, 99–115. doi: 10.1016/j.chembiol.2012.01.003 22284358 PMC3503453

[B56] Kitamura A. Maekawa Y. Uehara H. Izumi K. Kawachi I. Nishizawa M.. (2011). A Mutation in the Immunoproteasome Subunit PSMB8 Causes Autoinflammation and Lipodystrophy in Humans. J. Clin. Invest. 121(10), 4150–4160. doi: 10.1172/JCI58414 21881205 PMC3195477

[B57] Kloetzel P. M. (2001). Antigen Processing by the Proteasome. Nat. Rev. Mol. Cell Biol. 2, 179–188. doi: 10.1038/35056572 11265247

[B58] Koguchi Y. Kohno J. Nishio M. Takahashi K. Okuda T. Ohnuki T.. (2000). TMC-95a, B, C, and D, Novel Proteasome Inhibitors Produced by Apiospora Montagnei Sacc. TC 1093. Taxonomy, Production, Isolation, and Biological Activities. J. Antibiot 53 (2), 105–109. 10.7164/antibiotics.53.105 10805568

[B59] Kopito R. R. (2000). Aggresomes, Inclusion Bodies and Protein Aggregation. Trends Cell Biol. 10 (12), 524–530. doi: 10.1016/S0962-8924(00)01852-3 11121744

[B60] Krishnan K. M. Williamson K. C. (2018). The Proteasome as a Target to Combat Malaria: Hits and Misses. Transl. Res. 198, 40–47. doi: 10.1016/j.trsl.2018.04.007 30009761 PMC6422032

[B61] Kuhn D. J. Chen Q. Voorhees P. M. Strader J. S. Shenk K. D. Sun C. M.. (2007). Potent Activity of Carfilzomib, a Novel, Irreversible Inhibitor of the Ubiquitin-Proteasome Pathway, Against Preclinical Models of Multiple Myeloma. Blood 110(9), 3281–3290. doi: 10.1182/blood-2007-01-065888 17591945 PMC2200918

[B62] Kumar S. K. Bensinger W. I. Zimmerman T. M. Reeder C. B. Berenson J. R. Berg D.. (2014). Phase 1 Study of Weekly Dosing With the Investigational Oral Proteasome Inhibitor Ixazomib in Relapsed/Refractory Multiple Myeloma. Blood 124 (7), 1047–1055. doi: 10.1182/blood-2014-01-548941 24904120 PMC4468583

[B63] Kumar S. Rajkumar S. V. (2008). Many Facets of Bortezomib Resistance/Susceptibility. Blood 112 (6), 2177–2178. doi: 10.1182/blood-2008-07-167767 18779399

[B64] LaMonte G. M. Almaliti J. Bibo-Verdugo B. Keller L. Zou B. Y. Yang J.. (2017). Development of a Potent Inhibitor of the Plasmodium Proteasome With Reduced Mammalian Toxicity. J. Med. Chem. 60, 6721–6732. doi: 10.1021/acs.jmedchem.7b00671 28696697 PMC5554889

[B65] Li H. Ponder E. L. Verdoes M. Asbjornsdottir K. H. Deu E. Edgington L. E.. (2012). Validation of the Proteasome as a Therapeutic Target in Plasmodium Using an Epoxyketone Inhibitor With Parasite-Specific Toxicity. Chem. Biol. 19(12), 1535–1545. doi: 10.1016/j.chembiol.2012.09.019 23142757 PMC3529830

[B66] Li H. van derLinden W. A. Verdoes M. Florea B. I. McAllister F. E. Govindaswamy . (2014). Assessing Subunit Dependency of the Plasmodium Proteasome Using Small Molecule Inhibitors and Active Site Probes. ACS Chem. Biol. 9, 1869–1876. doi: 10.1021/cb5001263 24918547 PMC4136710

[B67] Li H. O’Donoghue A. J. van derLinden W. A. Xie S. C. Yoo E. Foe I. T.. (2016). Structure- and Function-Based Design of Plasmodium-Selective Proteasome Inhibitors. Nature 530, 233–236. doi: 10.1038/nature16936 26863983 PMC4755332

[B68] Lin Z. Chen X. Li Z. Zhou Y. Fang Z. Luo Y.. (2018). The Role of Bortezomib in Newly Diagnosed Diffuse Large B Cell Lymphoma: A Meta-Analysis. Ann. Hematol. 97 (11), 2137–2144. doi: 10.1007/s00277-018-3435-1 30027435

[B69] Lin G. Chidawanyika T. Tsu C. Warrier T. Vaubourgeix J. Blackburn C.. (2013). N,C-Capped Dipeptides With Selectivity for Mycobacterial Proteasome Over Human Proteasomes: Role of S3 and S1 Binding Pockets. J. Am. Chem. Soc 135 (27), 9968–9971. doi: 10.1021/ja400021x 23782398 PMC3773049

[B70] Lin G. Li D. Chidawanyika T. Nathan C. Li H. (2010). Fellutamide B is a Potent Inhibitor of the Mycobacterium Tuberculosis Proteasome. Arch. Biochem. Biophys. 501 (2), 214–220. doi: 10.1016/j.abb.2010.06.009 20558127 PMC2930046

[B71] Lin G. Li D. de Carvalho L. Deng H. Tao H. Vogt G.. (2009). Inhibitors Selective for Mycobacterial Versus Human Proteasomes. Nature 461, 621–626. doi: 10.1038/nature08357 19759536 PMC3172082

[B72] Liu C. Y. Kaufman R. J. (2003). The Unfolded Protein Response. J. Cell Sci. 116, 1861–1862. doi: 10.1242/jcs.00408 12692187

[B73] Löwe J. Stock D. Jap B. Zwickl P. Baumeister W. Huber R.. (2015). Crystal Structure of the 20*S* Proteasome From the Archaeon *T. Acidophilum* at 3.4 Å Resolution 268, 533–535. 10.1126/science.7725097 7725097

[B74] Majumder P. Rudack T. Beck F. Danev R. Pfeifer G. Nagy I.. (2019). Cryo-EM Structures of the Archaeal PAN-Proteasome Reveal an Around-the-Ring ATPase Cycle. Proc. Natl. Acad. Sci. U.S.A. 116, 534–539. doi: 10.1073/pnas.1817752116 30559193 PMC6329974

[B75] Makioka A. Kumagai M. Ohtomo H. Kobayashi S. Takeuchi T.. (2002). Effect of Proteasome Inhibitors on the Growth, Encystation, and Excystation of Entamoeba Histolytica and Entamoeba Invadens. Parasitol. Res. 88 (5), 454–459. doi: 10.1007/s00436-002-0601-z 12049464

[B76] Matthews W. Driscoll J. J. Tanaka K. Ichihara A. Goldberg A. L.. (1989). Involvement of the Proteasome in Various Degradative Processes in Mammalian Cells. Proc. Natl. Acad. Sci. U.S.A. 86, 2597–2601. doi: 10.1073/pnas.86.8.2597 2539595 PMC286964

[B77] Maupin-Furlow J. (2012). Proteasomes and Protein Conjugation Across Domains of Life. Nat. Rev. Microbiol. 10, 100–111. doi: 10.1038/nrmicro2696 PMC3291102 22183254

[B78] McDermott A. Jacks J. Kessler M. Emanuel P. D. Gao L. (2015). Proteasome-Associated Autoinflammatory Syndromes: Advances in Pathogeneses, Clinical Presentations, Diagnosis, and Management. Int. J. Dermatol. 54 (2), 121–129. doi: 10.1111/ijd.12695 25521013

[B79] Moreau P. Masszi T. Grzasko N. Bahlis N. J. Hansson M. Pour L.. (2016). Oral Ixazomib, Lenalidomide, and Dexamethasone for Multiple Myeloma. N Engl. J. Med. 374 (17), 1621–1634. doi: 10.1056/NEJMoa1516282 27119237

[B80] Moscvin M. Ho M. Bianchi G. (2021). Overcoming Drug Resistance by Targeting Protein Homeostasis in Multiple Myeloma. Cancer Drug Resist. 4, 1028–1046. doi: 10.20517/cdr.2021.93 35265794 PMC8903187

[B81] Muchamuel T. M. Anderl J. Fan A. Johnson H. Kirk C. Lowe E.. (2018). Kzr-616, a Selective Inhibitor of the Immunoproteasome, Blocks the Disease Progression in Multiple Models of Systemic Lupus Erythematosus (SLE). Ann. Rheumatic Dis. 77, 685. doi: 10.1136/annrheumdis-2018-eular.1100

[B82] Munshi N. C. Avet-Loiseau H. Rawstron A. C. Owen R.G. Child J.A. Thakurta A.. (2017). Association of Minimal Residual Disease With Superior Survival Outcomes in Patients With Multiple Myeloma: A Meta-Analysis 3, 1, 28–35. doi: 10.1001/jamaoncol.2016.3160 PMC5943640 27632282

[B83] Ng C. L. Fidock D. A. Bogyo M. (2017). Protein Degradation Systems as Antimalarial Therapeutic Targets. Trends Parasitol. 33 (9), 731–743. doi: 10.1016/j.pt.2017.05.009 28688800 PMC5656264

[B84] Olzmann J. A. Li L. Chin L. S. (2008). Aggresome Formation and Neurodegenerative Diseases: Therapeutic Implications. Curr. Med. Chem. 15 (1), 47–60. doi: 10.2174/092986708783330692 18220762 PMC4403008

[B85] Orlowski R. Z. Kuhn D. J. (2008). Proteasome Inhibitors in Cancer Therapy: Lessons From the First Decade. *Clin* . Cancer Res. 14, 1649–1657. doi: 10.1158/1078-0432.CCR-07-2218 18347166

[B86] Palumbo A. Anderson K. (2011). Multiple Myeloma. New Engl. J. Med. 364 (11), 1046–1060. doi: 10.1056/NEJMra1011442 21410373

[B87] Park J. E. Miller Z. Jun Y. Lee W. Kim K. B. (2018). Next-Generation Proteasome Inhibitors for Cancer Therapy. Transl. Res. 198, 1–16. doi: 10.1016/j.trsl.2018.03.002 29654740 PMC6151281

[B88] Paugam A. Bulteau A. L. Dupouy-Camet J. Creuzet C. Friguet B.. (2003). Characterization role protozoan parasite proteasomes Trends Parasitol. 19 (2), 55–59. 12586468 10.1016/s1471-4922(02)00064-8

[B89] Phillips M. Burrows J. Manyando C. van Huijsduijnen R. H. Van Voorhis W. C. Wells T. N.C.. (2017). Malaria. Nat. Rev. Dis. Primers 3, 17050. doi: 10.1038/nrdp.2017.50 28770814

[B90] Poli M. C. Ebstein F. Nicholas S. K. De Guzman M. M. Forbes L. R. Chinn I. K.. (2018). Heterozygous Truncating Variants in POMP Escape Nonsense-Mediated Decay and Cause a Unique Immune Dysregulatory Syndrome. Am. J. Hum. Genet. 102, 1126–1142. doi: 10.1016/j.ajhg.2018.04.010 29805043 PMC5992134

[B91] Rajkumar S. V. (2020). Multiple Myeloma: 2020 Update on Diagnosis, Risk-Stratification and Management. Am. J. Hematol. 95 (5), 548–567. doi: 10.1002/ajh.25791 32212178

[B92] Ray K. Harris H. (1985). Purification of Neutral Lens Endopeptidase: Close Similarities to a Neutral Proteinase in Pituitary. Proc. Natl. Acad. Sci. U.S.A. 82, 7545–7549. doi: 10.1073/pnas.82.22.7545 3906648 PMC390853

[B93] Reid E. G. Looney D. Maldarelli F. Noy A. Henry D. Aboulafia D.. (2018). Safety and Efficacy of an Oncolytic Viral Strategy Using Bortezomib With ICE/R in Relapsed/Refractory HIV-Positive Lymphomas. Blood Advances 2 (24), 3618–3626. doi: 10.1182/bloodadvances.2018022095 30573564 PMC6306883

[B94] Robak T. Huang H. Jin J. Zhu J. Liu T. Samoilova O.. (2015). LYM-3002 Investigators Bortezomib-Based Therapy for Newly Diagnosed Mantle-Cell Lymphoma. N Engl. J. Med. 372, 944–953. doi: 10.1056/NEJMoa1412096 25738670

[B95] Robertson C. D. (1999). The Leishmania Mexicana Proteasome. Mol. Biochem. Parasitol. 103 (1), 49–60. doi: 10.1016/S0166-6851(99)00110-3 10514080

[B96] Sanchez G. A. M. Reinhardt A. Ramsey S. Wittkowski H. Hashkes P. J. Berkun Y.. (2018). JAK1/2 Inhibition With Baricitinib in the Treatment of Autoinflammatory Interferonopathies. J. Clin. Invest. 128 (7), 3041–3052. doi: 10.1172/JCI98814 29649002 PMC6026004

[B97] Schmidt C. Berger T. Groettrup M. Basler M. (2018). Immunoproteasome Inhibition Impairs T and B Cell Activation by Restraining ERK Signaling and Proteostasis. Front. Immunol. 9, 2386. doi: 10.3389/fimmu.2018.02386 30416500 PMC6212513

[B98] Schroder M. Kaufman R. J. (2005). The Mammalian Unfolded Protein Response. Annu. Rev. Biochem. 74, 739–789. doi: 10.1146/annurev.biochem.73.011303.074134 15952902

[B99] Shaw M. K. He C. Y. Roos D. S. Tilney L. G. (2000). Proteasome Inhibitors Block Intracellular Growth and Replication of Toxoplasma Gondii. Parasitology 121 (1), 35–47. doi: 10.1017/S0031182099006071 11085223

[B100] Stokes B. H. Yoo E. Murithi J. M. Luth M. R. Afanasyev P. da Fonseca P. C.A.. (2019). Covalent Plasmodium Falciparum-Selective Proteasome Inhibitors Exhibit a Low Propensity for Generating Resistance *In Vitro* and Synergize With Multiple Antimalarial Agents. PloS Pathog. 15 (6), e1007722. doi: 10.1371/journal.ppat.1007722 31170268 PMC6553790

[B101] Studencka-Turski M. Çetin G. Junker H. Ebstein F. Krüger E. (2019). Molecular Insight Into the IRE1α-Mediated Type I Interferon Response Induced by Proteasome Impairment in Myeloid Cells of the Brain. Front. Immunol. 10. 10.3389/fimmu.2019.02900 PMC6932173 31921161

[B102] Totaro K. A. Barthelme D. Simpson P. T. Jiang X. Lin G. Nathan C. F.. (2017). Rational Design of Selective and Bioactive Inhibitors of the Mycobacterium Tuberculosis Proteasome. ACS Infect. Dis. 3 (2), 176–181. doi: 10.1021/acsinfecdis.6b00172 28183185 PMC5410965

[B103] Tschan S. Brouwer A. J. Werkhoven P. R. Jonker A. M. Wagner L. Knittel S.. (2013). Broad-Spectrum Antimalarial Activity of Peptido Sulfonyl Fluorides, a New Class of Proteasome Inhibitors. Antimicrob. Agents Chemother. 57 (8), 3576–3584. doi: 10.1128/AAC.00742-12 23689711 PMC3719782

[B104] Tyagi R. Srivastava M. Jain P. Pandey R. P. Asthana S. Kumar D.. (2022). Development of Potential Proteasome Inhibitors Against Mycobacterium Tuberculosis. J. Biomol Struct. Dyn 40 (5), 2189–2203. doi: 10.1080/07391102.2020.1835722 33074049

[B105] van de Donk N. W. C. J. Pawlyn C. Yong K. L. (2021). Multiple Myeloma. Lancet 397 (10272), 410–427. doi: 10.1016/S0140-6736(21)00135-5 33516340

[B106] Van de Pluijim R. W. Imwong M. Chau N. H. Hoa N. T. Thuy-Nhien N. T. Thanh N. V.. (2019). Determinants of Dihydroartemisinin-Piperaquine Treatment Failure in Plasmodium Falciparum Malaria in Cambodia, Thailand, and Vietnam: A Prospective Clinical, Pharmacological, and Genetic Study. Lancet Infect. Dis. 19, 952–961. doi: 10.1016/S1473-3099(19)30391-3 31345710 PMC6715822

[B107] Verhoeven D. Schonenberg-Meinema D. Ebstein F. Papendorf J. J. Baars P. A. van Leeuwen E. M.M.. (2022). Hematopoietic Stem Cell Transplantation in a Patient With Proteasome-Associated Autoinflammatory Syndrome (PRAAS). J. Allergy Clin. Immunol. 149 (3), 1120–27.e1128. doi: 10.1016/j.jaci.2021.07.039 34416217

[B108] Voges D. Zwickl P. Baumeister W. (1999). The 26S Proteasome: A Molecular Machine Designed for Controlled Proteolysis. Annu. Rev. Biochem. 68, 1015–1068. doi: 10.1146/annurev.biochem.68.1.1015 10872471

[B109] Wilk S. Orlowski M. (1980). Cation-Sensitive Neutral Endopeptidase: Isolation and Specificity of the Bovine Pituitary Enzyme. J. Neurochem. 35 (5), 1172–1182. doi: 10.1111/j.1471-4159.1980.tb07873.x 6778972

[B110] Wilk S. Orlowski M. (1983). Evidence That Pituitary Cation-Sensitive Neutral Endopeptidase is a Multicatalytic Protease Complex. J. Neurochem. 40 (3), 842–849. doi: 10.1111/j.1471-4159.1983.tb08056.x 6338156

[B111] Woodle E. S. Tremblay S. Brailey P. Girnita A. Alloway R. R. Aronow B.. (2020). Proteasomal Adaptations Underlying Carfilzomib-Resistance in Human Bone Marrow Plasma Cells 20, 399–410. doi: 10.1111/ajt.15634 PMC6984988 31595669

[B112] Woodle E. S. Tremblay S. Driscoll J. J. (2017). Targeting Plasma Cells With Proteasome Inhibitors: Principles From Primates. Jour Amer Soc Nephrol 28, 1951–1953. doi: 10.1681/ASN.2017040443 28592425 PMC5491302

[B113] World Health Organization (2020) World Malaria Report 2020 (World Health Organization) (Accessed March 23, 2022).

[B114] Xie S. C. Gillett D. L. Spillman N. J. Tsu C. Luth M. R. Ottilie S.. (2018). Target Validation and Identification of Novel Boronate Inhibitors of the Plasmodium Falciparum Proteasome. J. Med. Chem. 61, 10053–10066. doi: 10.1021/acs.jmedchem.8b01161 30373366 PMC6257627

[B115] Xie S. C. Metcalfe R. D. Mizutani H. Puhalovich T. Hanssen E. Morton C. J.. (2021). Design of Proteasome Inhibitors With Oral Efficacy *In Vivo* Against *Plasmodium Falciparum* and Selectivity Over the Human Proteasome. Proc. Natl. Acad. Sci. U.S.A. 118 (39), e2107213118. doi: 10.1073/pnas.2107213118 34548400 PMC8488693

[B116] Xi J. Zhuang R. Kong L. He R. Zhu H. Zhang J.. (2019). Immunoproteasome-Selective Inhibitors: An Overview of Recent Developments as Potential Drugs for Hematologic Malignancies and Autoimmune Diseases. Eur. J. Med. Chem. 182, 111646. doi: 10.1016/j.ejmech.2019.111646 31521028

[B117] Yoo E. Stokes B. H. de Jong H. Vanaerschot M. Kumar T. Lawrence N.. (2018). Defining the Determinants of Specificity of Plasmodium Proteasome Inhibitors. J. Am. Chem. Soc 140 (36), 11424–11437. doi: 10.1021/jacs.8b06656 30107725 PMC6407133

[B118] Zani B. Gathu M. Donegan S. Olliaro P. L. Sinclair D.. (2014). Dihydroartemisinin-Piperaquine for Treating Uncomplicated Plasmodium Falciparum Malaria. Cochrane Database Syst. Rev. 2014(1), CD010927. doi: 10.1002/14651858.CD010927 PMC4470355 24443033

[B119] Zhan W. Zhang H. Ginn J. Leung A. Liu Y. J. Michino M.. (2021). Development of a Highly Selective Plasmodium Falciparum Proteasome Inhibitor With Anti-Malaria Activity in Humanized Mice. Angew Chem. Int. Ed. Engl. 60, 9279–9283. doi: 10.1002/anie.202015845 33433953 PMC8087158

[B120] Zhang H. Lin G. (2021). Microbial Proteasomes as Drug Targets. PloS Pathog. 17 (12), e1010058. doi: 10.1371/journal.ppat.1010058 34882737 PMC8659679

[B121] Zhang L. Zhang M. Cao X. Zhou D. B. Fajgenbaum D. C. Dong Y. J.. (2020). A Prospective, Multicenter Study of Bortezomib, Cyclophosphamide, and Dexamethasone in Relapsed/Refractory iMCD. Leukemia Lymphoma 63, 618–626. doi: 10.1080/10428194.2021.1999437 35100929

[B122] Zhan W. Hsu H. C. Morgan T. Ouellette T. Burns-Huang K. Hara R.. (2019). Selective Phenylimidazole-Based Inhibitors of the *Mycobacterium Tuberculosis* Proteasome. J. Med. Chem. 62 (20), 9246–9253. doi: 10.1021/acs.jmedchem.9b01187 31560200 PMC7091493

